# Frequency and Genetic Determinants of Tigecycline Resistance in Clinically Isolated *Stenotrophomonas maltophilia* in Beijing, China

**DOI:** 10.3389/fmicb.2018.00549

**Published:** 2018-03-26

**Authors:** Jin Zhao, Yunxi Liu, Yi Liu, Dong Wang, Wentao Ni, Rui Wang, Youning Liu, Bo Zhang

**Affiliations:** ^1^Department of Pulmonary and Critical Care Medicine, Air Force General Hospital of PLA, Beijing, China; ^2^Department of Infection Management and Disease Control, Chinese PLA General Hospital, Beijing, China; ^3^Department of Respiratory Medicine, Chinese PLA General Hospital, Beijing, China; ^4^Department of Clinical Pharmacology, Chinese PLA General Hospital, Beijing, China

**Keywords:** *Stenotrophomonas maltophilia*, tigecycline, resistance, susceptibility, efflux pump, *smeDEF*

## Abstract

*Stenotrophomonas maltophilia* is an emerging nosocomial pathogen with high resistance to most clinically used antimicrobials. Tigecycline is a potential alternative antimicrobial for *S. maltophilia* infection treatment, but its resistance mechanism in clinical isolates is not fully elucidated. We investigated the antimicrobial susceptibility of 450 *S. maltophilia* isolated during 2012–2015 from three university hospitals in Beijing, China. These strains exhibited high susceptibility to minocycline (98.44%), sulfamethoxazole/trimethoprim (87.56%), tigecycline (77.78 %), doxycycline (81.33%), levofloxacin (67.56%), and ticarcillin/clavulanate (73.00%). The susceptibility of tigecycline-nonsusceptible strains (TNS) to doxycycline and levofloxacin was much lower than that of tigecycline-susceptible strains (TSS) (25.00% vs. 97.71% for doxycycline, *P* < 0.001; 17.00% vs. 82.00% for levofloxacin, *P* < 0.001). We further selected 48 TNS and TSS and compared the detection rate of eight tetracycline-specific genes by PCR and the expression level of six intrinsic multidrug resistance efflux pumps by real-time PCR. Only one *tetB* and two *tetH* genes in TNS and three *tetH* genes in TSS were detected, and the detection rate had no difference. The average expression level of *smeD* in TNS was higher than that in TSS [20.59 (11.53, 112.54) vs. 2.07 (0.80, 4.96), *P* < 0.001], while the average expression levels of *smeA*, *smeI*, *smeO*, *smeV*, and *smrA* were not significantly different, indicating that *smeDEF* was the predominant resistance genetic determinant in clinical *S. maltophilia*. Higher *smeD* expression was also observed in levofloxacin- and doxycycline-nonsusceptible isolates than in their corresponding susceptible isolates [16.46 (5.83, 102.24) vs. 2.72 (0.80, 6.25) for doxycycline, *P* < 0.001; 19.69 (8.07, 115.10) vs. 3.01(1.00, 6.03), *P* < 0.001], indicating that *smeDEF* was also the resistance genetic determinant to levofloxacin and doxycycline. The consistent resistance profile and common resistance genetic determinant highlight the importance of rational use of tigecycline for preventing the occurrence and spread of multidrug resistance.

## Introduction

*Stenotrophomonas maltophilia* is classified by the World Health Organization as one of the leading multidrug resistant organisms in hospital settings, and an increasing infection rate has been reported in worldwide and nationwide surveillance studies during the past 15 years (Fluit et al., 2001; Brooke, 2012, 2014; Sader et al., 2013; Walkty et al., 2014; Chang et al., 2015). Treatment of *S. maltophilia* infections is limited due to the extensive resistance displayed to most clinically used antimicrobials, and sulfamethoxazole/trimethoprim (SXT) is the only recommended first-line antimicrobial. However, its use is limited by a high incidence of allergic reactions, intolerance, and increasing resistance mediated by the spread of *sul1* and *sul2* genes (Toleman et al., 2007; Hu et al., 2011; Chang et al., 2015; Zhao et al., 2016). Tigecycline has been reported to retain good *in vitro* activity against *S. maltophilia* in worldwide surveillance and multicenter studies, and Wei et al. (2016) reported that 80.4% of clinical isolates in China, and 72.7% of SXT-resistant isolates were susceptible to tigecycline, making it a promising alternative antimicrobial for infection treatment (Stein and Babinchak, 2013; Rizek et al., 2015; Wei et al., 2015, 2016). Clinical studies revealed the similar effectiveness of tigecycline and SXT in the treatment of nosocomial *S. maltophilia* infection (Tekce et al., 2012; Wu and Shao, 2014). Therefore, tigecycline could be used for treatment of *S. maltophilia* infection, but invalid use and overuse of tigecycline might result in emergence and spread of resistance during treatment. At present, the tigecycline resistance mechanism in clinical isolates is unclear, and epidemiological surveys focused on frequency and genetic determinants of tigecycline resistance are needed in clinical isolates of *S. maltophilia*. These epidemiologic data could help to guide the appropriate use of tigecycline and preventing the occurrence and spread of multidrug resistance.

Resistance to tetracycline in Gram-negative bacteria is usually attributed to two different mechanisms: acquisition of tetracycline-specific resistance genes and increased expression of intrinsic multidrug resistance efflux pumps (Bartha et al., 2011; Nguyen et al., 2014; Chang et al., 2015; Sharma et al., 2016). For tetracycline-specific resistance genes, carriage of the flavin-dependent monooxygenase genes (*tetX*, *tetX1*, *tetX2*) could confer resistance against tigecycline (Bartha et al., 2011). The *tetX1* gene has been detected in tigecycline-nonsusceptible *Acinetobacter baumannii* isolates in China, indicating that the *tetX1* gene might play a role in the reduced tigecycline susceptibility in clinical *A. baumannii* isolates (Deng et al., 2014). The other tetracycline-specific genes, including that encoding for efflux pumps (*tetA*, *tetB*, *tetH*, *tet39*) and ribosomal protection proteins (*tetM*), were also commonly detected in Gram-negative bacteria, but their detection rate and potential role in mediating tigecycline resistance in *S. maltophilia* has not been investigated (Bartha et al., 2011; Nguyen et al., 2014; Chang et al., 2015). A variety of intrinsic efflux pumps were presented in *S. maltophilia* and the resistance-nodulation-cell division (RND) family were recognized as the most important intrinsic multidrug-resistance efflux pumps (Chang et al., 2015). The K279a sequence carries nine RND-type efflux pump genes (Crossman et al., 2008). Six of these efflux pumps have been characterized, among which SmeDEF, SmeIJK, SmeOP-ToICsm, and SmeVWX can confer resistance to tigecycline or tetracycline (Alonso and Martinez, 2000; Zhang et al., 2001; Cho et al., 2012; Gould et al., 2013; Lin et al., 2014; Chang et al., 2015; Sanchez and Martinez, 2015). Besides these RND efflux pumps, SmrA, a member of the ATP binding cassette family, confers resistance to fluoroquinolones and tetracycline (Al-Hamad et al., 2009). However, most of these studies were performed by genetic introduction, deletion, or mutation in a single reference or clinical strain, and could not be representative of the common clinical scenario. Though all of these intrinsic efflux pumps could mediate tigecycline resistance, which is the predominant genetic determinant in clinical strains is still unclear. Therefore, molecular epidemiological investigations on the tigecycline resistance genetic determinants in clinically isolated *S. maltophilia* are still needed.

To this end, we investigated the frequency and antimicrobial susceptibility of 450 clinical isolates of *S. maltophilia* obtained between 2012 and 2015 from three university hospitals in Beijing, China, and further explored the possible genetic determinants of tigecycline resistance. We compared the detection rate of various tetracycline-specific resistance genes by PCR and the expression levels of multidrug-resistance efflux pumps by real-time PCR in tigecycline-nonsusceptible strains (TNS, minimum inhibitory concentration [MIC] for tigecycline > 2 mg/L) vs. tigecycline-susceptible strains (TSS, MIC for tigecycline ≤ 2 mg/L). The epidemiological investigation of the resistance profile and resistance genetic determinants to tigecycline could aid in the rational use of tigecycline and help in the development of targeted prevention measures to contain the occurrence and spread of multidrug resistance.

## Materials and Methods

### Bacterial Strains and Susceptibility Test

During 2012 to 2015, 450 clinical *S. maltophilia* isolates were collected from hospitalized patients at Peking Union Medical College Hospital, the Chinese PLA General Hospital, and the Air Force General Hospital in Beijing, China. Tigecycline is classified as a special-use antibacterial drug in China and is approved only for the treatment of complicated skin and soft-tissue infections, complicated intra-abdominal infections, and bacterial pneumonia. These *S. maltophilia* strains were all isolated from respiratory tract specimens of patients with pulmonary infection. Bacterial species identification was performed using a Vitek II bacterial identification system (bioMérieux, Marcy-l’Étoile, France), and the results were further confirmed by species-specific PCR (Whitby et al., 2000). MIC results for SXT, minocycline, levofloxacin, ticarcillin/clavulanate, ceftazidime, and chloramphenicol were performed by the agar dilution method, and the results were interpreted according to the breakpoints suggested by CLSI (2015). Since there were no specified breakpoints criterion of tigecycline and doxycycline for *S. maltophilia*, tigecycline susceptibility breakpoint criterion for *Enterobacteriaceae* and doxycycline susceptibility breakpoint criterion for *Acinetobacter* spp. were used. *Escherichia coli* ATCC 25922 and *Pseudomonas aeruginosa* ATCC 27853 purchased from the American Type Culture Collection (Manassas, VA, United States) were used as quality control strains. SXT, minocycline, levofloxacin, ticarcillin/clavulanate, ceftazidime, and chloramphenicol were purchased from the National Institute for the Control of Pharmaceutical and Biological Products (Beijing, China). Tigecycline and moxifloxacin were obtained from Wyeth Pharmaceutical (Philadelphia, PA, United States) and Bayer Healthcare (Bayer Pharma AG, Wuppertal, Germany), respectively. Mueller-Hinton agar was purchased from Becton Dickinson and Co. (Franklin Lakes, NJ, United States). Antimicrobial solutions were prepared fresh on the day of use as previously described (CLSI, 2015).

### Detection of Tetracycline-Specific Genes

Of the 450 isolates detected, 48 TNS and 48 TSS *S. maltophilia* were selected randomly and screened by PCR reaction for the presence of eight tetracycline-specific resistance genes (*tetA*, *tetB*, *tetM*, *tetH*, *tet39*, *tetX*, *tetX1*, and *tetX2*) using specific primers (**Table 1**) and Taq DNA Polymerase (TaKaRa Bio, Tokyo, Japan). PCR cycling parameters were as follows: initial activation at 95°C for 5 min; 30 amplification cycles at 95°C for 30 s, 55°C for 30 s, and 72°C for 30 s; and a final extension step at 72°C for 7 min. A 50-μL reaction volume was used, and the products were separated by 1% (w/v) agarose gel electrophoresis (Amresco, Solon, OH, United States) followed by ethidium bromide staining. Images were acquired using a Gel Doc^TM^ EQ imaging system (Bio-Rad, Hercules, CA, United States).

**Table 1:** Primers used for specific amplification of tetracycline-specific resistance genes and intrinsic multidrug-resistance efflux pumps of *S. maltophilia.*

Primer name	Sequence (5′–3′)	Origin	Purpose
tetA-F	CGTAATTCTGAGCACTGTC	^∗^Access number: AY196695	PCR for *tetA*
tetA-R	GTTGCATGATGAAGAAGACC		
tetB-F	GTGGAACTGACAACTTGTC	^∗^Access number: JN247441	PCR for *tetB*
tetB-R	CACTCAGTATTCCAAGCCT		
tetH-F	GCTGATCACCGTATTAGATG	^∗^Access number: 210972275	PCR for *tetH*
tetH-R	TGTCCTATTGGCAACAAGC		
tetM-F	CAAGCTATATCCTACAGCGA	^∗^Access number: JF830611	PCR for *tetM*
tetM-R	GCATACAGATATTCTCTGGA		
tet39-F	CTCCTTCTCTATTGTGGCTA	^∗^Access number: EU495993	PCR for *tet39*
tet39-R	ATCCTGCCCATAGATAACC		
tetQ-F	TAACCGAGAATCTGCTGTT	^∗^Access number: 807867	PCR for *tetQ*
tetQ-R	CGTCCAACAACTCATTGATA		
tetX1-F	TCAGGACAAGAAGCAATGAA	#Bartha et al., 2011	PCR for *tetX1*
tetX1-R	TATTTCGGGGTTGTCAAACT		
tetX2-F^†^	TTAGCCTTACCAATGGGTGT	#Bartha et al., 2011	PCR for *tetX, X2*^†^
tetX2-R^†^	CAAATCTGCTGTTTCACTCG		
sme 27	TGCCAGCGACAGTGCAAAGGGTC	#Sanchez et al., 2002	PCR for *smeT* and smeD/smeT intergenic region
sme 43	CCAGGATCATCGATCTGCC		
smeA-F	GTCGACCTGGTACAGCA	^∗^Access number: AM743169	qRT-PCR for *smeA*
smeA-R	ACCTTAACCTGTGCCTTG		
smeD-F	CGGTCAGCATCCTGATGGA	#Garcia-Leon et al., 2014	qRT-PCR for *smeD*
smeD-R	TCAACGCTGACTTCGGAGAACT		
smeI-F	ACTGCGATGAACACCGTTACC	#Garcia-Leon et al., 2014	qRT-PCR for *smeI*
smeI-R	CACGTCACCCTGCTTCACTTC		
smeO-F	CAGGAAAGTCCACTGTCGTTC	#Garcia-Leon et al., 2014	qRT-PCR for *smeO*
smeO-R	CACGTCGCCCTTCTTCAC		
smeW-F	GCCCACACCATCTCGTTCCC	#Chen et al., 2011	qRT-PCR for *smeW*
smeW-R	TAGCCGTTGCCGTTGCCC		
smrA-F	TGGAAGTGGCGATGTTCGAT	^∗^Access number: FJ481984	qRT-PCR for *smrA*
smrA-R	CATGGCGCTTGAAGAAGTCG		
16SrDNA-F	GACCTTGCGCGATTGAATG	#Chen et al., 2011	qRT-PCR for *smeA*
16SrDNA-R	CGGATCGTCGCCTTGGT		

*qRT-PCR, quantitative real-time PCR.*^∗^*These primers were designed in this study and the gene sequence access number of reference strain were presented.*^*#*^*These primers were cited from other studies and the reference information were presented in reference section. aaaTetX2-F and tetX2-R could amplify both tetX and tetX2 genes.*

### Detection of Expression of Intrinsic Multidrug Efflux Pumps

The expression levels of six multidrug efflux pump genes (*smeA*, *smeD*, *smeI*, *smeO*, *smeV*, and *smrA*) were assessed by real-time PCR using specific primers (**Table 1**). *S. maltophilia* suspensions were prepared and inoculated in Mueller-Hinton broth (Difco, Cockeysville, MD, United States). After overnight culture, total DNase-treated RNA was extracted using the RNeasy kit (Tiangen, Beijing, China). cDNA was synthesized using the primeScript RT-PCR kit (TaKaRa Bio) following the manufacturer’s instructions. Real-time PCR was performed using the DNA Engine Opticon 2 real-time PCR detection system (Bio-Rad) and a SYBR premix EX Taq kit (TaKaRa Bio). Thermocycling conditions were as follows: Taq activation at 95°C for 5 s, followed by 40 cycles at 95°C for 15 s and 58°C for 30 s. Each real-time PCR reaction was conducted in triplicate. Analysis of real-time PCR results was carried out by the 2^-ΔΔC_T_^ method. The housekeeping gene 16S rDNA was used as an internal control to normalize the expression of target genes and a susceptible *S. maltophilia* from Air Force General Hospital (K106, MIC_tigecycline_ = 0.25 mg/L) was used as an external control to normalize the relative expression levels of target genes from clinical isolates. All real-time PCR experiment amplifying the target gene should also include real-time PCR reaction amplifying 16S rDNA gene for both test strains and external control K106 in the same plate. The *C*_t_ values for the 16S rRNA gene in K106 were all within one cycle for each experiment under all conditions tested.

### Amplification for *smeT* and *smeT/D* Intergenic Region

To identify the sequence variations of the *smeT* and the *smeT/D* intergenic region, PCR reactions were performed using primers *sme* 27 and *sme* 43 presented by Sanchez et al. (2002; **Table 1**). The primers were designed for sequence amplification of the *smeT* and the *smeT/D* intergenic region in D457R (accession number: AJ316010). The *S. maltophilia* K279a and ATCC13637 were used as control to ensure the accuracy of the PCR reaction. Sequence of the *smeT* and the *smeT/D* intergenic region of D457R were blasted in NCBI database^1^. All sequence of *smeT* gene and *smeT/D* intergenic region presented in NCBI database were downloaded and aligned in Mega 6.

### Statistical Analyses

SPSS 22.0 (SPSS Inc., Chicago, IL, United States) was used for statistical analyses. Comparative analyses of antimicrobial susceptibility rate and detection rate of tetracycline-specific resistant genes were performed by the Chi-squared test or Fisher’s exact test, as appropriate. Expression levels of intrinsic multidrug-resistance efflux pumps are presented as median (interquartile range) and compared by Mann-Whitney *U* test. All comparisons were two-tailed, and *P* < 0.05 was considered to indicate statistically significant differences.

## Results

### Antimicrobial Susceptibility Profiles

Tigecycline exhibited good activity (MIC_50_/MIC_90_: 1/8 mg/L, susceptibility rate: 77.78%) (**Table 2**). SXT (87.56%), minocycline (98.44%), and ticarcillin/clavulanate (76.00%) retained high *in vitro* activity against clinical isolates of *S. maltophilia*, while ceftazidime (26.44%) and chloramphenicol (12.00%) exhibited poor activity. TSS strains displayed high susceptibility to minocycline (100.00%), SXT (89.71%), doxycycline (97.71%), levofloxacin (82.00%), and ticarcillin/clavulanate (76.86%), while the TNS strains displayed high susceptibility to minocycline (94.00%), SXT (80.00%), and ticarcillin/clavulanate (73.00%), but poor susceptibility to doxycycline (25.00%) and levofloxacin (17.00%). The susceptibility rate of TNS strains to doxycycline and levofloxacin was much lower than that of TSS strains (25.00% vs. 97.71% for doxycycline, *P* < 0.001; 17.00% vs. 82.00% for levofloxacin, *P* < 0.001).

**Table 2:** *In vitro* susceptibility of 450 clinical isolates of *S. maltophilia* to eight clinically used antimicrobials.

		Total (*N* = 450)				TNS (*N* = 100)					TSS (*N* = 350)					
Antimicrobial agents	MIC_range_	MIC_50_/ MIC_90_	*S*	*I*	*R*	MIC_range_	MIC_50_/ MIC_90_	*S*	*I*	*R*	MIC_range_	MIC_50_/ MIC_90_	*S*	*I*	*R*	*P*
TIG^†^	0.25–32	1/8	77.78	8.67	13.56											
MIN	0.125–16	0.5/2	98.44	0.89	0.67	1–16	2/4	94.00	4.00	2.00	0.125–2	0.5/1	100.00	0.00	0.00	<0.001
DOX^#^	0.5 ≥ 32	2/8	81.33	14.22	4.44	4 ≥ 32	8/16	25.00	61.00	14.00	0.5–32	2/4	97.71	0.86	4.83	<0.001
LEV	0.125 ≥ 32	2/16	67.56	11.33	21.11	1 ≥ 32	8/32	17.00	17.00	66.00	0.125–32	2/4	82.00	9.71	24.02	<0.001
T/K	1 ≥ 128	8/64	76.00	15.78	8.22	1 ≥ 128	8/128	73.00	14.00	13.00	1 ≥ 128	8/64	76.86	16.29	20.49	0.426
CET	2 ≥ 64	64/>64	26.44	9.33	64.22	2 ≥ 64	64/ > 64	25.00	7.00	68.00	2 ≥ 64	64/>64	26.86	10.00	85.71	0.710
CHL	1 ≥ 64	16/64	12.00	38.00	50.00	8 ≥ 64	32/128	1.00	11.00	88.00	1 ≥ 64	16/64	15.14	45.71	69.24	<0.001
SXT	(2.273/0.125)/(>152/8)	(4.75/0.25)/(152/8)	87.56		12.44	(4.75/0.25)/(>152/8)	(4.75/0.25)/(>152/8)	80.00		20.00	(2.273/0.125)/(>152/8)	(4.75/0.25)/(76/4)	89.71		28.64	0.009

*MIC, minimum inhibitory concentration; TNS, tigecycline-nonsusceptible S. maltophilia; TSS, tigecycline-susceptible S. maltophilia; S, susceptible; I, intermediate; R, resistant; CET, ceftazidime; CHL, chloramphenicol; DOX, doxycycline; LEV, levofloxacin; MIN, minocycline; T/K, ticarcillin/clavulanate; TIG, tigecycline; SXT, sulfamethoxazole/trimethoprim. aaaMIC breakpoint criteria of these antimicrobials for S. maltophilia refer to susceptible breakpoint criteria for Enterobacteriaceae published by CLSI (2015); ^#^MIC breakpoint criteria of these antimicrobials for S. maltophilia refer to susceptible breakpoint criteria for Acinetobacter spp. published by CLSI (2015).*

### Detection of Tetracycline-Specific Genes

We screened eight tetracycline-specific resistance genes (*tetA*, *tetB, tetM, tetH, tet39, tetX, tetX1*, and *tetX2*) in 48 selected clinical TNS and 48 TSS clinical *S. maltophilia* by routine PCR analysis. Only one *tetB* and two *tetH* genes in TNS and three *tetH* genes in TSS were detected, and the detection rate was not different between the two groups.

### Expression of Intrinsic Multidrug Efflux Pumps

**FIGURE 1:**
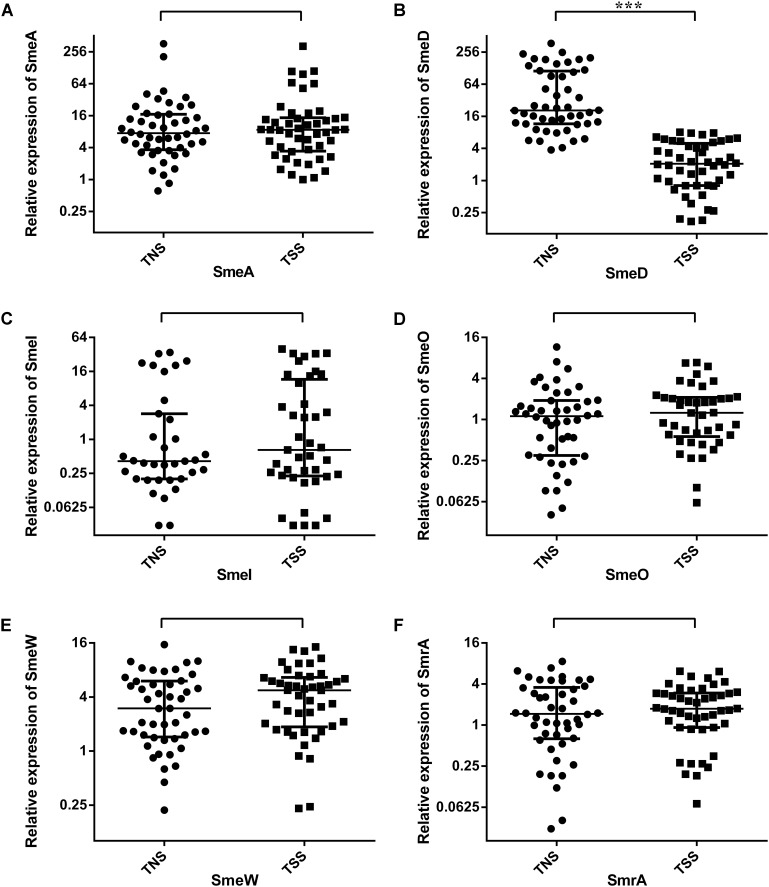
Real-time PCR analysis of intrinsic multidrug-resistance efflux pumps gene expression profiles in tigecycline-nonsusceptible and tigecycline-susceptible *S. maltophilia*. **(A–F)** Represent the relative expression level of *smeA, smeD, smeI, smeO, smeW*, and *smrA*, respectively. TNS, tigecycline-nonsusceptible *S. maltophilia*; TSS, tigecycline-susceptible *S. maltophilia*. Each dot corresponds to a clinical isolate. Expression levels of intrinsic multidrug-resistance efflux pumps are presented as median (interquartile range) and compared by non-parametric tests. ^∗∗∗^*P* < 0.001.

Real-time PCR showed that the average relative expression levels [median (interquartile range)] of *smeA*, *smeD*, *smeI*, *smeO*, *smeW*, and *smrA* in TNS *S. maltophilia* were 7.47 (3.64, 16.93), 20.59 (11.53, 112.54), 0.41 (0.20, 2.85), 1.12 (0.30, 1.90), 2.99 (1.44, 6.03), and 1.46 (0.63, 3.57), respectively. The values in TSS *S. maltophilia* were 8.64 (3.42, 14.68), 2.07 (0.80, 4.96), 0.65 (0.23, 11.61), 1.26 (0.56, 2.10), 4.75 (1.86, 6.61), and 1.74 (0.92, 2.96), respectively. Quantitative analysis (**Figure 1**) revealed that the average expression level of *smeD* was higher in the TNS group than in the TSS group [20.59 (11.53, 112.54) vs. 2.07 (0.80, 4.96), *P* < 0.001]. We further divided the 98 selected *S. maltophilia* into levofloxacin-susceptible and levofloxacin-nonsusceptible strains (including 47 LSS and 49 LNS), doxycycline-susceptible and doxycycline-nonsusceptible strains (including 50 DSS and 46 DNS). Compared with that in the corresponding LSS and DSS, the expression of *smeD* was higher in LNS [16.46 (5.83, 102.24) vs. 2.72 (0.80, 6.25), *P* < 0.001] and DNS [19.69 (8.07, 115.10) vs. 3.01(1.00, 6.03), *P* < 0.001] (**Table 3**).

**Table 3:** Expression levels of intrinsic multidrug-resistance efflux pumps genes in *S. maltophilia* nonsusceptible to doxycycline or levofloxacin and the corresponding susceptible controls.

Efflux pump genes	DOX			LEV		
	DNS	DSS	*P*	LNS	LSS	*P*
*smeA*	7.14 (3.86, 15.1)	8.99 (3.47, 17.79)	0.477	7.11 (3.52, 14.76)	9.21 (3.66, 17.98)	0.343
*smeD*	19.69 (8.07, 115.1)	3.01 (1.00, 6.03)	<0.001	16.46 (5.83, 102.24)	2.72 (0.8, 6.25)	<0.001
*smeI*	0.43 (0.24, 1.68)	0.65 (0.18, 14.37)	0.797	0.42 (0.24, 2.85)	0.51 (0.2, 11.61)	0.843
*smeO*	1.15 (0.46, 2.01)	1.29 (0.46, 2.05)	0.726	1.12 (0.42, 1.82)	1.29 (0.47, 2.14)	0.495
*smeW*	3.51 (1.63, 5.79)	4.75 (1.64, 6.52)	0.511	3.65 (1.64, 6.3)	4.74 (1.63, 6.48)	0.742
*smrA*	1.46 (0.63, 3.47)	1.74 (0.88, 3.01)	0.458	1.46 (0.55, 3.49)	1.74 (0.95, 2.9)	0.458

*DOX, doxycycline; DNS, doxycycline-nonsusceptible S. maltophilia; DSS, doxycycline-susceptible S. maltophilia; LEV, levofloxacin; LNS, levofloxacin-nonsusceptible S. maltophilia; LSS, levofloxacin-susceptible S. maltophilia. Numbers denote median values and interquartile ranges.*

### Amplification for *smeT* and *smeT/D* Intergenic Region

Primers *sme* 27 and *sme* 43, which designed for amplification of D457R failed to amplify the target gene in most of our clinical strains, and even the K279a and ATCC13637. The sequence of D457R (accession number: AJ316010) had 90–100% identity in *smeT* gene and 83–100% identity in *smeT/D* intergenic region with sequences of *S. maltophilia* strains from NCBI database. There was a variety of sequence variants located on the *smeT* and *smeT/D* intergenic region, including original and terminal region of these genes (see Supplementary Material).

## Discussion

*Stenotrophomonas maltophilia* is one of the leading multidrug resistant organisms in hospital settings and can cause severe nosocomial respiratory and bloodstream infections (Looney et al., 2009; Brooke, 2014; Chang et al., 2015). Tigecycline is regarded as a potential alternative antimicrobial for infection treatment (Looney et al., 2009; Chang et al., 2015). Clinical isolates of *S. maltophilia* in Beijing showed good *in vitro* susceptibility to tigecycline. The tigecycline susceptibility rate in Beijing was lower than that reported in the Taiwan Surveillance of Antimicrobial Resistance Study from 1998 to 2008 and in the countries in the SENTRY Antimicrobial Surveillance Program from 2009 to 2012, but similar to that reported in SENTRY from 2011 to 2014 (Wu et al., 2012; Sader et al., 2014, 2016). The difference might be partially due to the different geographic origins of the sources and the timing of the studies. More selective pressure caused by the gradual increase in the use of tigecycline in recent years might also contribute to the lower susceptibility rate. The clinical *S. maltophilia* isolates from Beijing showed high *in vitro* susceptibility to minocycline, SXT, and ticarcillin/clavulanate, rendering these compounds appropriate antimicrobial treatments for *S. maltophilia* infection in this area. Ceftazidime and chloramphenicol had poor activity, and they should not be used as empirical treatment. The susceptibility rate against doxycycline and levofloxacin in TNS *S. maltophilia* was much lower than that in the TSS group. Highly consistent antimicrobial resistance profiles against tigecycline, doxycycline, and levofloxacin indicated that they might share common genetic resistance determinants, and this criterion should be taken into account when deciding on the treatment regimen.

Due to the global spread of carbapenem-resistant *A. baumannii*, KPC-producing *K. pneumoniae*, and SXT-resistant *S. maltophilia*, tigecycline is regarded as the last resort in the control of clinical infections (Tekce et al., 2012; Sun et al., 2013; Deng et al., 2014; Chang et al., 2015; He et al., 2015). Epidemiological investigations have demonstrated that overexpression of the RND efflux pump AdeABC plays the predominant role on the tigecycline resistance in clinically isolated *A. baumannii*, while the RND efflux pump AdeIJK and flavin-dependent monooxygenase TetX1 are also involved in tigecycline resistance (Deng et al., 2014; Li et al., 2015). In *K. pneumonia*, the RND efflux pump AcrAB contributes to tigecycline resistance in clinical isolates (Bratu et al., 2009; He et al., 2015). Tigecycline is not effective against *Pseudomonas aeruginosa*, and the reduced susceptibility is attributed to RND efflux pump MexXY (Dean et al., 2003; Sun et al., 2013). However, the tigecycline resistance mechanism in clinical isolates has not been fully elucidated. To identify the predominant genetic determinants of tigecycline resistance in clinical isolates of *S. maltophilia*, we compared the detection rate of eight tetracycline-specific resistance genes and the expression levels of six multidrug-resistance efflux pump genes. Among the eight tetracycline-specific resistance genes, only a few *tetB* or *tetH* genes were detected in clinical *S. maltophilia* isolates, and the detection rate was not different between the *TNS* and *TSS* groups, indicating that the tetracycline-specific resistance genes were not the predominant tigecycline resistance mechanism.

Regarding the intrinsic efflux pump genes, a variety of efflux pumps have been shown to be associated with resistance to tigecycline or tetracycline by the genetic introduction, deletion, or mutation in a single reference or clinical strain, including SmeDEF, SmeIJK, SmeOP-TolCsm, SmeVW, and SmrA (Alonso and Martinez, 2000; Zhang et al., 2001; Al-Hamad et al., 2009; Cho et al., 2012; Gould et al., 2013; Lin et al., 2014; Sanchez and Martinez, 2015). Considering the common resistance profile between tigecycline and levofloxacin, we also detected the expression level of *smeABC*, which could mediate the extrusion of quinolones (Li et al., 2002). Quantitative analysis revealed that the average expression level of *smeD* was higher in TNS *S. maltophilia* than in TSS strains, while the remaining five intrinsic multidrug efflux genes did not show significant differences. The results indicate that overexpression of *smeDEF* is the predominant tigecycline resistance mechanism of clinical *S. maltophilia* isolates in Beijing. SmeDEF was identified as a novel multidrug efflux pump from *S. maltophilia* in 2000. The pump is formed by an inner membrane protein (SmeE), an outer membrane protein (SmeF), and a membrane fusion protein (SmeD) (Alonso and Martinez, 2000). Zhang et al. (2001) have identified tigecycline as a substrate of SmeDEF in both intrinsic and acquired resistance by genetic deletion and mutation of ULA-511 strain. We confirmed that overexpression of *smeDEF* is the common genetic determinants of resistance to tigecycline in clinical isolates of *S. maltophilia* in Beijing. Subsequent studies after Zhang et al. (2001) demonstrated that this pump mediated not only multiple resistance to a variety of structurally unrelated antimicrobial agents, but also a number of disinfectants and heavy-metal ions (Sanchez et al., 2002; Gould and Avison, 2006; Chang et al., 2015). A battery of epidemiological investigations have indicated that quinolone resistance in clinical isolates of *S. maltophilia* is associated with overexpression of *smeDEF*, and the extrusion activity of SmeDEF is not abolished by the efflux pump inhibitor Phe-Arg-β-naphthylamide (Sanchez et al., 2002; Jia et al., 2015; Chong et al., 2017). We further compared the expression levels of intrinsic efflux pumps between levofloxacin-nonsensitive and levofloxacin-sensitive, and doxycycline-nonsensitive and doxycycline-sensitive groups. The results revealed a higher expression of *smeD* in levofloxacin- and doxycycline-nonsensitive isolates than in their corresponding sensitive counterparts, which demonstrated that overexpression of SmeDEF is also the common genetic determinants of multiple resistance to doxycycline, and levofloxacin.

Our results highlight the importance of the rational use of antibiotics to prevent the emergence and spread of multidrug resistance. Due to a variety of intrinsic and acquired antimicrobial resistance mechanisms, therapeutic options for *S. maltophilia* treatment are limited, especially for patients who cannot tolerate SXT. Tigecycline, doxycycline, and levofloxacin have become the few available potential alternative antimicrobial agents when SXT is unsuitable for the treatment of *S. maltophilia* owing to contraindications or resistance. However, inappropriate use and overuse of tigecycline might result in the occurrence and spread of tigecycline, and also the emergence of resistance to levofloxacin and doxycycline. A serendipitous mutation in the regulatory *smeT* gene, such as A/T mutation at position 498, could lead to overexpression of *smeDEF* and multiple resistances to tigecycline, doxycycline, and levofloxacin (Sanchez et al., 2002). If clinical strains with this mutation were selected and enriched by pressure of prolonged exposure to subinhibitory concentrations, the selection and enrichment would result in emergence of multidrug resistance and the failure of clinical treatment. Using primers *sme* 27 and *sme* 43 presented by Sanchez et al. (2002), we tried to identify other sequence variations located on the *smeT* and the *smeT/D* intergenic region in clinical *S. maltophilia*, but the primers designed for amplification of D457R failed to amplify most of our clinical strains, and even the K279a and ATCC13637. We further downloaded all of the sequence of *smeT* and the *smeT/D* intergenic region in NCBI database and performed sequence alignment. The result of alignment revealed a variety of sequence variants located on the *smeT* and *smeT/D* intergenic region, including original and terminal region of these genes, therefore it was difficult to design general primers that could amplify the complete sequence of *smeT* in clinical *S. maltophilia* from different areas and times. Further researches are still needed to identify the possible sequence variations of *smeT* gene leading to the overexpression of *smeDEF* in clinical scenario.

## Conclusion

Clinical isolates of *S. maltophilia* in Beijing showed good *in vitro* susceptibility to tigecycline. *S. maltophilia* had highly consistent antimicrobial resistance profiles against tigecycline, doxycycline, and levofloxacin, and the *smeDEF* efflux pumps gene was the common genetic determinant of resistance to these three drugs. The consistent resistance profile and common resistance genetic determinant highlight the importance of rational use of tigecycline for preventing the occurrence and spread of multidrug resistance.

## Author Contributions

YNL and BZ conceived and designed the experiments. JZ and YXL executed the experiments. JZ, YL, and DW performed and wrote the manuscript. RW and WN helped to edit the manuscript.

## Conflict of Interest Statement

The authors declare that the research was conducted in the absence of any commercial or financial relationships that could be construed as a potential conflict of interest.

## Footnotes

1 https://blast.ncbi.nlm.nih.gov/Blast.cgi?PROGRAM=blastn&PAGE_TYPE=BlastSearch&LINK_LOC=blasthome
