# Supplementary material for: Frequency and Genetic Determinants of Tigecycline Resistance in Clinically Isolated Stenotrophomonas maltophilia in Beijing, China
Source: Front Microbiol. 2018 Mar 26;9:549. doi: 10.3389/fmicb.2018.00549 (PMC5879106; doi:10.3389/fmicb.2018.00549)
Supplement: Supplementary file 1 [file Data_Sheet_1.DOCX]

**Figure 1. Sequence alignment of all sequence of *smeT* gene presented in NCBI database in Mega 6.**

**A: Result of alignment from sequence 1 to 99;**

**B: Result of alignment from sequence 548 to 660.**

**Figure 2. Sequence alignment of all sequence of *smeT/D* intergenic region presented in NCBI database in Mega 6.**

**A: Result of alignment from sequence 1 to 136;**

**B: Result of alignment from sequence 99 to 233.**
