# Supplementary figures and images for: Frequency and Genetic Determinants of Tigecycline Resistance in Clinically Isolated Stenotrophomonas maltophilia in Beijing, China
Source: Front Microbiol. 2018 Mar 26;9:549. doi: 10.3389/fmicb.2018.00549 (PMC5879106; doi:10.3389/fmicb.2018.00549)

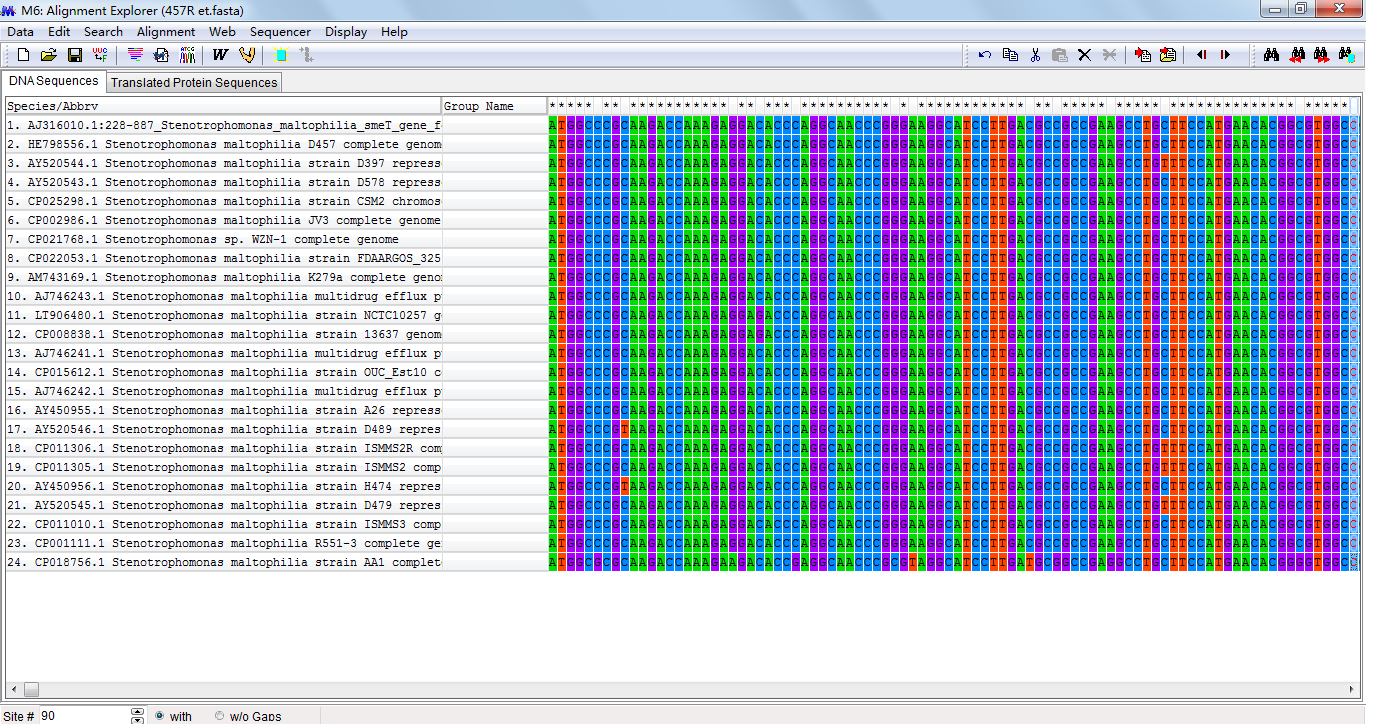

Supplement: Supplementary file 2 [file Image_1.TIF]

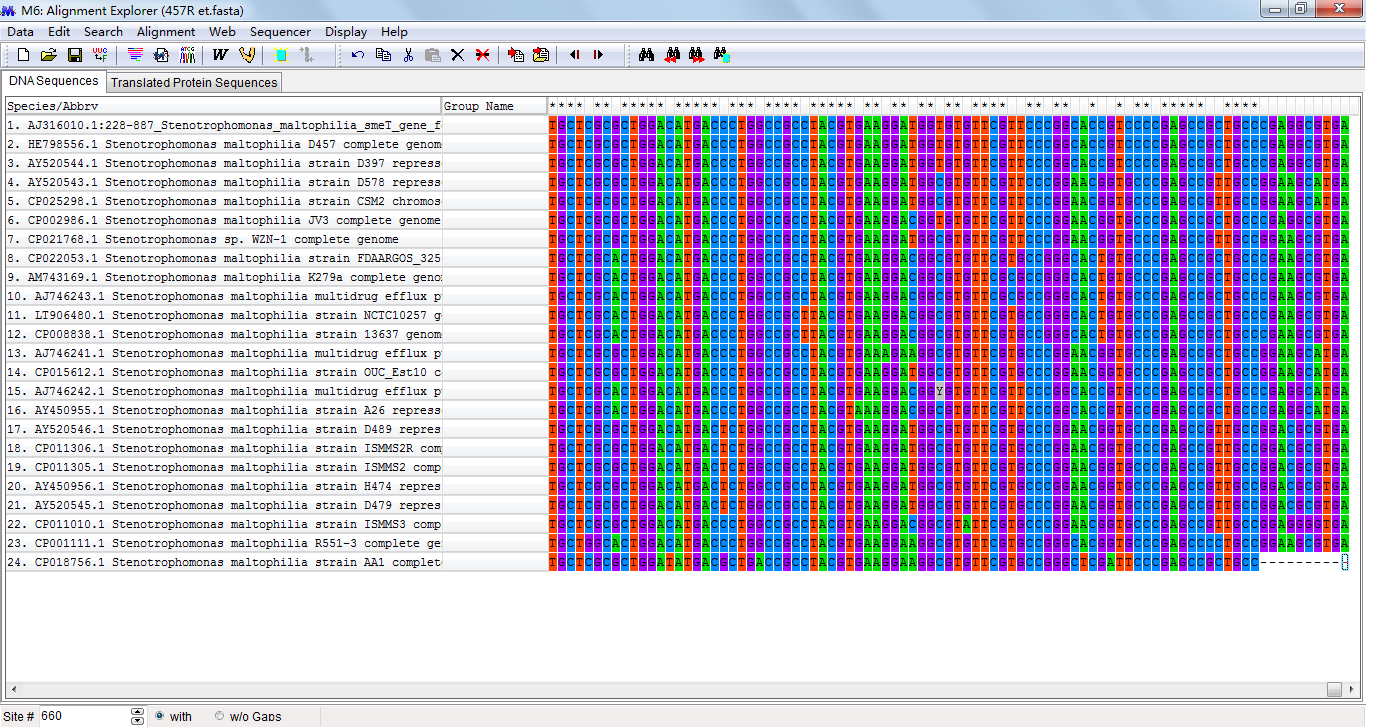

Supplement: Supplementary file 3 [file Image_2.TIF]

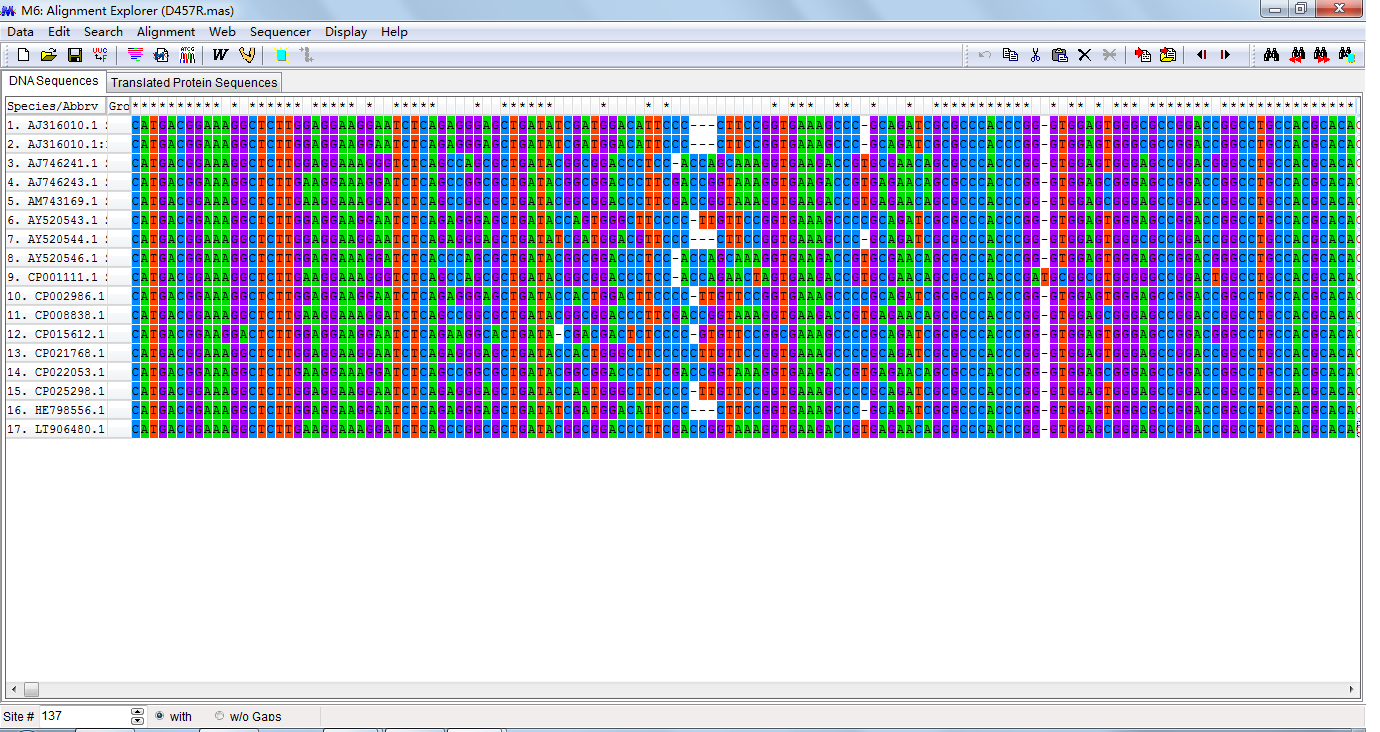

Supplement: Supplementary file 4 [file Image_3.TIF]

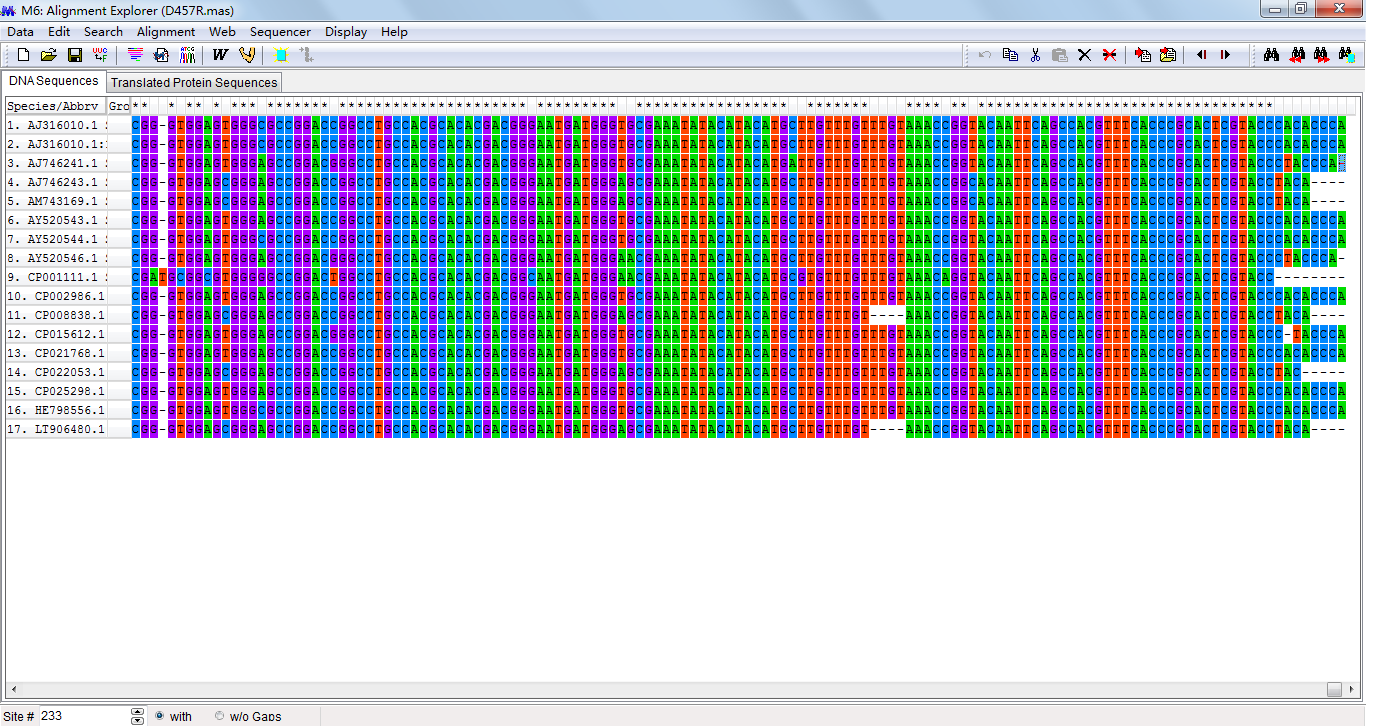

Supplement: Supplementary file 5 [file Image_4.TIF]
